# Evaluation of health-related knowledge, attitudes, and behaviors of undergraduate students by cardiovascular risk factors

**DOI:** 10.1017/S1463423621000578

**Published:** 2021-10-14

**Authors:** Volkan Aydin, Caner Vizdiklar, Ahmet Akici, Mehmet Akman, Dilek Gogas Yavuz, Zehra Aysun Altikardes, S. Guniz Kucukguzel, Mumine Topcu, Berrin Aysevinc, Ali Serdar Fak

**Affiliations:** 1 Marmara University Hypertension and Atherosclerosis Research Center (HIPAM), Istanbul, Turkey; 2 Department of Medical Pharmacology, Istanbul Medipol University International School of Medicine, Istanbul, Turkey; 3 Department of Medical Pharmacology, Marmara University School of Medicine, Istanbul, Turkey; 4 Department of Pharmacy Services, Vocational School of Health Services, Fenerbahce University, Istanbul, Turkey

**Keywords:** cardiovascular risk, healthy lifestyle, smoking, young adult

## Abstract

**Aim:**

To determine the presence of cardiovascular (CV) risk (CVR) factors in university students and evaluate how these factors are affected from the knowledge, attitudes, and habits of the individuals regarding healthy lifestyle.

**Background:**

Starting from early ages, lifestyle habits such as lack of physical activity, unhealthy eating, and inappropriate drug use increase CV and metabolic risks of individuals.

**Methods:**

In April–May 2018, sociodemographic characteristics of 770 undergraduate students, in addition to their knowledge, attitudes, and habits regarding their nutrition and physical activity status were obtained through face-to-face questionnaires. CVR factors were determined according to blood pressure, blood glucose, total cholesterol levels, and anthropometric measurements. Collected data were compared by CVR factor presence (CV[+] or CV[−]) in students.

**Findings:**

The mean age of the participants was 22.3 ± 2.6 years. 59.6% were female and 71.5% were students of non-health sciences. In total, 274 individuals (35.9%) belonged to CV(+) group (mean risk number: 1.3 ± 0.5) with higher frequency in males (42.1% versus 31.6%, *P* < 0.05). The most common CVR factors were smoking (20.6%), high total cholesterol (7.5%), and hypertension/high blood pressure (6.0%). 15.5% of the participants regularly used at least one drug/non-pharmaceutical product. 11.3% complied the Mediterranean diet well. 21.9% of CV(+) stated consuming fast food at lunch compared to 14.3% of CV(−) (*P* < 0.05). 44.6% stated exercising below the CV-protective level.

**Conclusions:**

This study showed one-third of university students was at CVR, independent of their sociodemographic characteristics. Furthermore, the students appear to perform below expectations in terms of nutrition and physical activity. Extensive additional measures are needed to encourage young individuals for healthy nutritional and physical activity habits.

## Introduction

Starting from early ages, sedentary lifestyle and unhealthy eating habits increase metabolic and cardiovascular (CV) risks (CVR) of individuals. These issues impose medical, economic, and psychological burden on the healthcare system, which might emerge as morbidity and mortality (Clark *et al.*, 2014). Moreover, conditions such as obesity and CV diseases might present earlier due to unhealthy lifestyle habits and environmental factors (Arora *et al.*, 2019, Fryar *et al.*, 2020). One of the most efficient strategies to prevent or minimize these public health issues is to define the risks and intervene appropriately as soon as possible (Nissinen *et al.*, 2001).

As for all age groups, healthy lifestyle is of key significance also in younger population. CV and metabolic risk factors presenting early are known to cause predisposition to long-term effects (Hardy *et al.*, 2015). In addition, results from existing literature show that effects of risk-modifying interventions might wear off in time, and younger individuals might comply poorly to intervention-induced lifestyle changes (Van De Vijver *et al.*, 2012, Tran and Sojobi 2020). Therefore, promoting healthy lifestyle to the young as soon as possible is important due to potential of better outcomes, such as effectively raising and spreading the much needed awareness, detecting possible health risks earlier and managing any related issues properly, thus reducing the medical and economic burden (Naderi *et al.*, 2012).

It could be expected that after end of a stressful period of preparation to entry exams, students recently admitted to university might comply with healthy lifestyle principles more than before. Also, a graduate who adopted a healthy lifestyle might possibly instill awareness to her/his surroundings (Vedanthan *et al.*, 2016). On the other hand, various issues concerning university students more closely might be threatening for their overall health. For example, students leaving their families for college might fail to properly manage the changes in their daily activities such as eating, accommodation and exercising, leading to medium to long-term health-related risks (Plotnikoff *et al.*, 2015). Apart from those, it should be noted that tobacco, recreational drugs, and various drug/non-pharmaceutical products, some claiming to promote bodybuilding and enhance performance, are frequently used by young individuals (İlhan *et al.*, 2016, Valentine *et al.*, 2018).

Considering all those, it is crucial to determine the extent of CVR burden in young population along with the affecting factors, in order to appropriately screen their risks and control them early. In that regard, this study aimed to determine the presence of CVR factors in university students and evaluate how these factors are affected from the knowledge, attitude, and habits of the individuals regarding healthy lifestyle.

## Methods

This cross-sectional study was conducted in April–May 2018 as part of Marmara University Student Healthy Lifestyle and Cardiovascular Risk Assessment (MUSAY-CARD) Project, coordinated by Marmara University Hypertension and Atherosclerosis Research Center (HIPAM). Data were collected from the volunteers at the previously determined locations in the campuses by the involved physicians and nurses using face-to-face questionnaires, physical examination, and blood tests. Written informed consent was obtained from each participant. Ethical approval was obtained from Marmara University School of Medicine Ethical Committee for Clinical Studies (approval number: 09.2017.327).

### Participants

The study population consisted of students of Marmara University, which provides education to more than 70000 students at various campuses in Istanbul. Participants of the study were determined via convenience sampling. Main campus and two other campuses, which were home to faculties of health sciences and sports sciences, were selected. All students studying at those campuses were informed about the study, and those who volunteered to participate answered the questionnaires and underwent physical examination and blood tests. A total of 1000 participants was determined as target sample group. Inclusion criteria were being an undergraduate student, being 18 years of age and older, and signing the informed consent form. Final year students, along with associate, graduate, and postgraduate students, were not included in the study.

### Clinical measurements

Body mass index (BMI) values of the participants were calculated according to height and weight measurements (Nuttall, 2015). Blood pressure (BP) measurements were made in accordance with current guideline recommendations (Williams *et al.*, 2018). Random total cholesterol (TC) and blood glucose (BG) measurements were done from capillary blood obtained by fingerstick. As test kits and strips, “Accutrend Plus/Cholesterol” (Roche Diagnostics, Basel, Switzerland) was used for TC and “Accu-Chek Performa” (Roche Diagnostics, Basel, Switzerland) was used for BG measurement.

### Questionnaire data

Knowledge, attitude, and habits of the participants about their nutrition and physical activity were assessed via questionnaires. The first form included 22 items, questioning sociodemographic characteristics, nutritional and physical activity levels, along with attitudes and behaviors regarding regular use of drugs and non-pharmaceutical medicinal products. Compliance with the Mediterranean diet was examined by using Mediterranean Diet Quality Index (KIDMED). KIDMED consists of a questionnaire with 16 ‘yes/no’ questions that focus on the participants’ eating habits to assess the compliance to Mediterranean style of diet, which is suggested to be suitable for a healthy lifestyle and lower CVR (Serra-Majem *et al.*, 2004). This index was reported to be valid and reliable in Turkish population (Şahingöz *et al.*, 2019). Students’ level of knowledge on CV diseases was measured using the Cardiovascular Disease Risk Factors Knowledge Level (CARRF-KL) Scale, which consists of 28 items questioning participants’ knowledge about characteristics of CV diseases, CVR factors, and outcome of changes in risk behaviors (Arikan *et al.*, 2009). Health literacy status was evaluated via responses to Turkish Health Literacy Survey (HLS-TR). HLS-TR, which is the Turkish adoptation of HLS-EU-Q47 (European Health Literacy Survey Questionnaire), is a 32-item, 5-point Likert scale used to evaluate health literacy status by answers to questions about accessing, understanding, appraising, and applying health-related information (Abacigil *et al.*, 2019). Collected data were compared according to the sociodemographic characteristics, and the risk factors were determined by measurements and answers. Answers to CARRF-KL scale were evaluated using 0–28 score range, whereas responses to KIDMED index and HLS-TR scale were analyzed categorically, with the first one as ‘poor’ and ‘average/good’, and the second as ‘adequate/excellent’ and ‘inadequate/problematic-limited’.

### Evaluation

Based on answers to questionnaires, physical examination findings, and measurements, individuals with at least one of the following characteristics were classified in ‘high-risk group’ for CV disease risk. Accordingly, those who reported a history of smoking or diagnosis of diabetes, hypertension, and/or another cardiac diagnosis, the individuals with high BP (≥140/90 mmHg), high BG (≥200 mg/dl), and/or high TC (≥200 mg/dl), those with increased waist circumference (WC, >102 cm for males and >88 cm for females) and BMI over 30 kg/m^2^ were included in high CVR [CV(+)] group. Individuals without those traits were classified as low risk [CV(−)]. Data collected on physical activity levels and eating habits were compared according to CVR presence and sociodemographic characteristics.

### Statistical analysis

Collected data were analyzed using IBM SPSS Statistics for Windows, Version 22.0 (IBM Corp., Armonk, NY, USA) software. Analyzed data were expressed as numbers, percentages, and/or mean ± standard deviation values, wherever appropriate. Frequency analysis was used for statistical evaluation, whereas chi-square test was used for comparison of the categorical variables. Continuous variables were evaluated for normal distribution prior to comparison using Kolmogorov–Smirnov analysis. Normally distributed data were compared using Student’s *t*-test, whereas Mann–Whitney *U* test was used in case of an absence of normality. An overall 5% type I error level was used to infer statistical significance.

## Results

A total of 770 students were included in the study (response rate: 77.0%). The mean age was 22.3 ± 2.6 (range: 19–41 years) and 59.6% (*n* = 459) were female. Details of sociodemographic characteristics and health-related habits were presented in Table 1. More than half (53.4%) of the students fell into ‘average/good’ KIDMED category, whereas in terms of health literacy, 51.8% were ‘inadequate/problematic-limited’ (Table 1). The mean CARRF-KL score was 22.8 ± 2.9 (Table 2).

**Table 1.** Sociodemographic and other characteristics of the participants

		*n*	%
Sex	Female	459	59.6
	Male	311	40.4
Field of study	Health related	215	28.5
	Non-health related	540	71.5
Year at undergraduate education	Preparatory class and first year students	291	40.2
	Second year students	233	32.2
	Third year students	168	23.2
	Fourth year students (and above)	31	4.3
Residence	With family	318	45.6
	With relatives	20	2.9
	With other students or alone	104	14.9
	At dormitory	242	34.7
	Others	13	1.8
Monthly income level of family	≥Minimum wage	174	25.2
	Minimum wage ×2	256	37.1
	Minimum wage ×3	156	22.6
	Minimum wage × ≥ 4	104	15.1
Maternal education level	Unqualified	86	12.3
	Primary/secondary school graduate	372	53.3
	High school graduate	138	19.8
	Undergraduate	102	14.6
Paternal education level	Unqualified	31	4.5
	Primary/secondary school graduate	304	43.7
	High school graduate	188	27.1
	Undergraduate	172	24.7
CVR^a^ factors	Yes	274	35.9
	No	490	64.1
Physical activity level	Sufficient physical activity	296	44.6
	Insufficient physical activity	368	55.4
Lunch habits	At dining hall	483	69.0
	Fast food at canteen	115	16.4
	Soup, salad etc. at canteen	21	3.0
	Sandwich etc. prepared at home	7	1.0
	Hot meal	29	4.1
	No lunch	30	4.3
History of weight-lowering diet	Yes	231	34.7
	No	435	65.3
KIDMED^b^	Good	85	11.3
	Average	320	42.3
	Poor	350	46.4
HLS-TR^c^	Inadequate	99	14.4
	Problematic-limited	258	37.4
	Adequate	205	29.8
	Excellent	127	18.4

a Cardiovascular risk.

b Mediterranean Diet Quality Index.

c Turkish Health Literacy Scale.

**Table 2.** Distribution of general characteristics by presence of cardiovascular risk

		CV(+)^a^		CV(−)^b^		Total		
		*n*	%	*n*	%	*n*	%	*P*-value
Sex	Female	144	31.6	311	68.3	455	100	0.003
	Male	130	42.1	179	57.9	309	100	
Field of study	Health related	56	26.2	158	73.8	214	100	0.001
	Non-health related	208	38.9	327	61.1	535	100	
Year of undergraduate education	Prep/first/second year	179	34.5	340	65.5	519	100	0.188
	Third year or later	81	39.7	123	60.3	204	100	
Residence	With family/relatives	104	31.1	230	68.9	334	100	0.208
	At dormitory/with other students/alone	128	35.7	231	64.3	359	100	
Monthly income level of family	≤×2 minimum wage	295	69.1	132	30.9	427	100	0.097
	>×2 minimum wage	96	37.1	163	62.9	259	100	
Maternal education level	Unqualified	20	23.3	66	76.7	86	100	0.072
	Undergraduate	36	35.3	66	64.7	102	100	
Paternal education level	Unqualified	8	25.8	23	74.2	31	100	0.156
	Undergraduate	67	39.2	104	60.8	171	100	
Sufficient physical activity	Yes	110	30.1	256	69.9	366	100	0.070
	No	108	36.7	186	63.3	294	100	
Eating habits	Fast food at lunch	49	43.0	65	57.0	114	100	0.016
	Others	178	31.3	390	68.7	568	100	
History of weight-lowering diet	Yes	93	40.8	135	59.2	228	100	0.006
	No	131	30.2	303	69.8	434	100	
KIDMED^c^ category	Poor	125	35.7	225	64.3	350	100	0.761
	Average/good	139	34.3	266	65.7	405	100	
HLS-TR^d^ category	Adequate/excellent	113	31.7	244	68.3	357	100	0.120
	Inadequate/problematic-limited	123	37.0	209	63.0	332	100	
CARRF-KL^e^ score		22.6 ± 3.0		22.9 ± 2.8		22.8 ± 2.9		0.251

a Cardiovascular high-risk group.

b Cardiovascular low-risk group.

c Mediterranean Diet Quality Index.

d Turkish Health Literacy Scale.

e Cardiovascular Risk Factors Knowledge Level Scale.

### Cardiovascular risk factors

**Figure 1.**
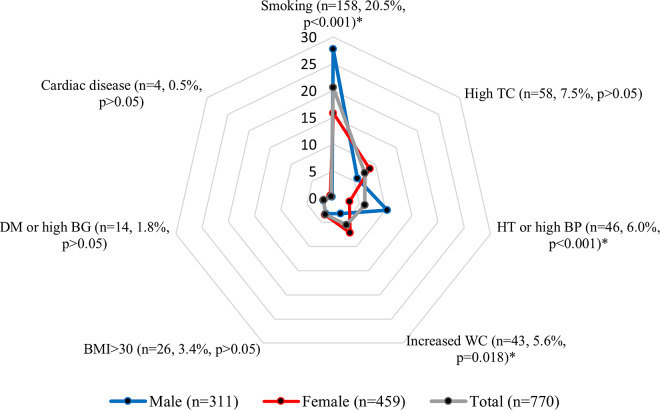
Percentage distribution of risk factors by gender. *: *P* < 0.05. TC: Total cholesterol, HT: Hypertension, BP: Blood pressure, BMI: Body mass index, DM: Diabetes mellitus, BG: Blood glucose.

Participants with single CVR factor was 27.5% (*n* = 210) of all, whereas 8.4% (*n* = 64) had multiple-risk factors. Mean risk count of the 274 CV(+) individuals (35.9%) was 1.3 ± 0.5 (range: 1–3). The most common risk factor was smoking (20.6%), followed by high TC (7.5%), hypertension/high BP (6.0%), increased WC (5.6%), obesity (3.4%), diabetes/high BG (1.8%), and cardiac disease (0.5%), respectively. The prevalence of smoking and hypertension/high BP was higher in males (27.7% versus 15.8% and 10.3% versus 3.1%, respectively; *P* < 0.001 for each), whereas increased WC was more common in females (7.2% versus 3.2%, respectively; *P* = 0.018) (Figure 1). Parametric values of the examined data by gender were presented in Supplementary Table 1.

The mean ages of CV(+) and CV(−) groups were similar (22.6 ± 3.0 and 22.2 ± 2.4, respectively; *P* > 0.05). CV(+) individuals were more common in males (42.1% versus 31.6% in females; *P* = 0.003). The percentage of non-health-related field students were higher in CV(+) group (78.8% versus 67.4% in CV(−); *P* = 0.001). CV(+) and CV(−) were similar in terms of other sociodemographic characteristics, CARRF-KL scores, KIDMED index, and HLS-TR categories (*P* > 0.05), (Table 2).

### Physical activity status

Almost half of the students (44.6%) declared their physical activity as below the CV-protective level. Inadequate physical activity was more widespread in females (53.4% versus 30.5% in males; *P* < 0.001). Physical activity level was not significantly different by CVR presence, category of residence, monthly income of the family, and maternal education level (*P* > 0.05). Distribution of participants stating an increase or decrease in activity after starting university were similar in CV(+) and CV(−) (*P* > 0.05), (Supplementary Table 2).

### Eating habits

Approximately one-sixth (16.5%) of the participants reported consuming fast foods at the university cafeteria for lunch. That was more common in CV(+) than CV(−) (21.9% versus 14.3%, respectively; *P* = 0.017). Distribution by gender was similar (*P* > 0.05). 33.8% of the students stated going on a diet at least once to lose weight, and the percentage of those were higher in CV(+) (41.5% versus 30.8% in CV(−), *P* = 0.006) and females (38.2% versus 29.2% in males, *P* = 0.03). Students who reported having a better diet since the start of university were more common in CV(−) group compared to CV(+) (27.2% versus 17.0%; *P* = 0.003). Conversely, percentage of participants stating that their diet had worsened were higher in CV(+) (49.4% versus 38.3%, respectively; *P* = 0.005), (Supplementary Table 3).

### Multiple cardiovascular risk factors

Of 64 students with multiple-risk factors, 53 (7.0% of all participants) had 2 and 11 (1.4%) had 3. Most commonly accompanying CVR factors were increased WC with obesity in 19 students (29.7%), high TC with smoking in 15 (23.4%), and increased WC with smoking in 10 (15.6%) (Table 3). Comparison of single versus multiple-risk factor individuals showed that history of weight-lowering diet was more commonly observed in single-risk group (*P* < 0.001), whereas “average/good” KIDMED category was more abundant in multiple-risk group (*P* = 0.019), (Supplementary Table 4).

**Table 3.** Distribution of cardiovascular risk factors among patients with multiple risks

	Smoking *n* (%)	High TC *n* (%)	HT/high BP *n* (%)	Increased WC *n* (%)	BMI > 30 *n* (%)	DM/high BG *n* (%)	Cardiac disease *n* (%)
Smoking		15 (23.4%)	9 (14.0%)	10 (15.6%)	5 (7.8%)	8 (12.5%)	1 (1.6%)
High TC	15 (23.4%)		2 (3.13%)	6 (9.4%)	3 (4.7%)	3 (4.7%)	0
HT/high BP	9 (14.0%)	2 (3.13%)		3 (4.7%)	1 (1.6%)	0	0
Increased WC	10 (15.6%)	6 (9.4%)	3 (4.7%)		19 (29.7%)	1 (1.6%)	0
BMI > 30	5 (7.8%)	3 (4.7%)	1 (1.6%)	19 (29.7%)		0	0
DM/high BG	8 (12.5%)	3 (4.7%)	0	1 (1.6%)	3 (4.7%)		0
Cardiac disease	1 (1.6%)	0	0	0	0	0	
Total^a^	48 (75.0%)	29 (45.3%)	15 (23.4%)	39 (60.95%)	31 (48.4%)	12 (18.8%)	1 (1.6%)

a Percentages were calculated over the total number of patients with multiple risks (*n* = 64).

### Regular use of drugs

Of all participants, 15.5% (*n* = 109) reported use of at least one drug/non-pharmaceutical product regularly. 9.1% of the students (*n* = 64) were regular drug users due to underlying chronic conditions. This was more common in CV(+) group (12.9% vs 7.1% in CV(−), *P* = 0.012) and females (11.9% versus 4.7% in males, *P* = 0.001). Those who declared regular use of non-pharmaceutical products (8.5%) were more common in females (10.0%) compared to males (4.7%), (*P* = 0.011); whereas the distribution by CV(+) and CV(−) groups was similar (10.3% versus 6.9%, respectively; *P* > 0.05). A total of 50 drugs containing 30 different active ingredients were reported. Most commonly used three drugs were levothyroxine (*n* = 9, 18% of all drugs), sertraline (*n* = 4, 8%), and acetylsalicylic acid (*n* = 3, 6%), whereas in non-pharmaceutical products, the first three consisted of vitamins (51.8%), dietary supplements (19.6%), and protein powder (12.5%).

## Discussion

This study involved high number (*n* = 770) of university students, focusing on their lifestyle habits regarding health and conducted with direct and voluntary participation of student clubs. In the study, which represented both genders in a balanced manner, remarkable findings related to the lifestyle and CVRs of the young population were revealed. Accordingly, the frequency of CVR factors among university students being similar to the general population might be regarded as concerning. Despite the partial relationship between CVRs of participants with physical activity levels and eating habits, health literacy levels and monthly income levels of families do not appear to be directly related with those risks.

Although the EuroSCORE risk model is more widely used in clinical practice nowadays, due to its validity being limited to individuals at age 40 or older, mainly Framingham Risk Analysis parameters were used in the study to assess CVR of our sample of younger age (Cooney *et al.*, 2009, Pencina *et al.*, 2009). It is noteworthy that more than one-fourth of participants have at least one CVR factor, and smoking was the leading one. According to Global Adult Tobacco Survey data, smoking prevalence in Turkey was 27.1% (Turkish Public Health Institution, 2014). In a study done among university students in seven European countries, smoking prevalence in Turkey was reported as 49.8% in males and 37.7% in females, whereas the rates in other countries vary between 19%–44% in males and 17%–39% in females (Pischke *et al.*, 2015). Percentage of regular smokers in our study is lower both in males (27.7%) and females (15.8%) than the aforementioned studies, hence, our results could suggest a downward trend. On the other hand, it should be noted that around three-fourth of the participants were first or second-year students. Previously, it was reported that prevalence of smoking doubled in senior undergraduate students compared to freshmen (Sarioglu *et al.*, 2016). Furthermore, any delay in age of onset of smoking due to early interventions is known to help in reducing nicotine addiction (Everett *et al.*, 1999). All these information suggest that despite the effective campaigns and the measures taken against tobacco products in Turkey in recent years, high prevalence of smoking among university students retains its place as a health issue. Thus, for further interventions planned against smoking among university population, focusing to the students those newly started to education might be more beneficial.

The second most common risk factor was high TC, which was similar in both genders. Turkish Chronic Diseases and Risk Factors Prevalence Study, which determined the frequency of various CV and metabolic risk factors nationally, reported mean TC values as 148.8 ± 0.9 mg/dl in 15–24-year-old males and 153.7 ± 0.8 mg/dl in females of the same age. That study also reported high cholesterol levels showed female predominance in all age groups, except 25–44-year-old age group (Turkish Public Health Institution, 2013). In our study, TC levels under 150 mg/dl could not be measured, hence, that prevents a direct comparison of our results with the existing literature. Nevertheless, mean TC values of both female (175.1 ± 21.7) and male (171.1 ± 22.4) subjects over 150 mg/dl seems to be consistent with the findings of previous studies. The prevalence of increased WC (7.2% in females versus 3.2% in males) observed in our study was lower than the national prevalence (10.6% in females versus 5.9% in males); however, these two results were in accordance in terms of female predominance (Turkish Public Health Institution, 2013). The similarity of university students and the general population in frequencies of modifiable risk factors such as smoking, TC, and WC needs attention. On the other hand, the rapid rise in CVR seen in females both in Turkey and worldwide in recent years made raising awareness necessary and stimulated the initiation of campaigns as a result (Maas *et al.*, 2011). Similar interventions might also be necessary and effective for young people, including university students.

Another remarkable finding of the study was the CV(+) and CV(−) groups being alike in terms of family income and parent education. Also, the distribution of CARRF-KL scores and HLS-TR categories of the two groups were similar. These findings point out that the family income and parent education levels, along with the health literacy of the participants, might not be directly reflected onto their health-related attitudes and behaviors. Although that approach of these young adults could change at their later ages, it was reported that undesirable effects of CVR factors occurring at an early age might present in adulthood, even if they were treated as soon as possible (Murthy *et al.*, 2016, Tran and Sojobi, 2020). For that reason, that holds a significant importance to develop effective and participatory ways to transform health-related knowledge into internalized attitudes and behaviors (Schwalm *et al.*, 2019, Tran and Sojobi, 2020).

Almost half of the subjects reported insufficient physical activity levels. More than half of females and approximately one-third of males had insufficient physical activity levels. These findings are consistent with the results of current literature, which emphasized low levels of physical activity in whole population including university students (Dinc *et al.*, 2015, Tek *et al.*, 2020). Higher percentage of individuals with increased physical activity (55.2%) than those who were more sedentary than before (27.5%) might be related to well-developed facilities in their campuses, as well as their previous exercising habits. In addition, significant reduction in physical activity after starting university education were reported in a recent study (Alkhateeb *et al.*, 2019). Relatively high numbers in individuals with increased physical activity could be regarded as favorable. On the other hand, students in the last years of high school education might have been away from exercising during long periods of exam preparation. Therefore, a significant proportion of students being physically more active could be attributed to them being excessively sedentary before university. Overall, despite slight improvements in some students, physical activity levels in university students could be regarded as below expectations. In accordance with the relevant medical guidelines, interventions facilitating and encouraging exercising should be chosen over instead of solely settling with preferences of the individuals (Piepoli *et al.*, 2016).

Another notable finding of the study was the higher rate of fast food consumption, which could be interpreted as an unfavorable eating behavior, in CV(+) group. Moreover, among those who declared such behavior, there were more subjects who stated a decrease in their physical activity levels. These findings are important in pointing out that attitudes and behaviors unfavorable for CV system might coexist, and holistic approaches are needed to provide effective solutions. Consistent with the student statements above, it was revealed that only 11% of the participants comply well with the Mediterranean diet according to KIDMED index. A study conducted among high school students in Istanbul reported that the rate of healthy eating behavior, which was described as consuming at least three of the food groups in the food pyramid at the right amounts, was found to be 23% (Akman *et al.*, 2012). This could suggest that dietary habits might become less heart friendly when transitioning from high school to university. Another study conducted among university students in UK reported that similar to our results, healthy eating behavior was found to be 19% and 31% of the participants were found to have risky or mixed eating habits (Tanton *et al.*, 2015). In a qualitative study conducted with university students, the most critical finding was put forth as the importance of consulting students while planning any interventions to develop healthy eating behaviors on campus (Sogari *et al.*, 2018). This result suggests that attempts to be made with the participation of university student groups, as in our study, might be more successful.

Approximately one out of every seven students reported regular drug/non-pharmaceutical product use, and one-eleventh of all participants declared regular drug use due to chronic disease. Levothyroxine being the most reported drug seems in accordance with the high prevalence of hypothyroidism in Turkey (Erdoǧan *et al.*, 2009). The second most used drug was the antidepressant drug, sertraline. The prominence of sertraline among antidepressants may be related to being among most frequently prescribed drugs by both adult and child and adolescent psychiatrists (Aydin *et al.*, 2020). That finding might imply the significance of depression and anxiety disorders in young adult group, which our subjects belong to. Also, one-twelfth of the students using at least one non-pharmaceutical product regularly is noteworthy. Vitamin, mineral, and protein supplements being the most used ones is consistent with the results of existing studies (Lieberman *et al.*, 2015). As a result of regular use of these products without supervision of a healthcare professional, undesirable effects such as overdose, medication errors, adverse effects, and interactions with other drugs were reported (Ruiz, 2010). For example, some studies stated excessive intake of protein powder, which was among the most commonly used non-pharmaceutical products in our study (Sánchez Oliver *et al.*, 2011). Uncontrolled use of these products was reported to lead unwanted outcomes such as deteriorated kidney and liver functions, musculoskeletal diseases, and rapid progression of coronary heart disease (Delimaris, 2013). It could be assumed that limiting the use of these products to the situations under the supervision of healthcare professionals might reduce the aforementioned risks.

Our findings should be interpreted with its limitations. Firstly, convenience sampling, which was used in the study, is not without flaws. As a non-probability sampling method, this could affect the generalizability of the results negatively, and might lead to biases emerging from the subjects’ tendencies that might differ from the population, for example, those opted to participate might have been more inclined to be aware of the principles of healthy lifestyle compared to a random individual. It should be taken into account that the answers of the participants, regarding their physical activity and eating habits etc., might not reflect their behavior in real life. Hunger/satiety state of the individuals, as well as recent changes in habits that may affect BG and TC values, were not questioned prior to clinical measurements. Also, TC values lower than 150 mg/dl were below the threshold of detection for the device. Thus, absolute numerical values of these individuals could not be obtained, and those results had to be excluded from parametric analyses. In addition, health-related field students participating in the study might have increased overall health literacy and CV knowledge scores, so that might have led to a bias. Finally, no detailed inquiries have been made regarding the use of illegal products such as illicit drugs. None of the participants reported such use as an answer to the question related to drug/non-pharmaceutical products. Thus, a lack of more detailed questioning of any illicit drug use might also be regarded as a limitation of the study.

In conclusion, this study revealed that whilst being more common in males, one out of every three university students had at least one CVR that might negatively affect their overall health. Despite the presence of CVR appearing not to be directly related to health literacy of participants, family income, and parent education levels, the prevalence of CV problems and risks being that of the general population seems concerning. Furthermore, the participants seem to perform below expected levels in terms of nutrition and physical activity, and unhealthy eating habits were more common among CV(+) subjects. These findings point out that in terms of preventive medicine, extensive additional measures are needed to encourage young individuals, especially the ones in at-risk groups, for healthy nutritional and physical activity habits. The outputs of the study could be regarded as examples to similar interventions that might be initiated in other campuses and universities to increase awareness of the students regarding healthy life, which has an emerging importance.

## Supplementary material

For supplementary material accompanying this paper visit https://doi.org/10.1017/S1463423621000578
